# Toroidal counter electrode for ionic propulsion

**DOI:** 10.1038/s41598-022-23377-5

**Published:** 2022-11-08

**Authors:** Marius Chirita, Adrian Ieta

**Affiliations:** 1 Applied Physics/Condensed Matter Department, National Institute for Research and Development in Electrochemistry and Condensed Matter, 300224 Timisoara, Romania; 2 Electrical and Computer Engineering Department, SUNY Oswego, Oswego, NY 13126-3599 USA

**Keywords:** Aerospace engineering, Electrical and electronic engineering, Mechanical engineering

## Abstract

Significant attention has recently been given to applications of ionic wind to atmospheric propulsion. Rotational ionic engines (RIE) have also demonstrated to have potential for in-atmosphere propulsion in negative polarity. However, such devices have not yet produced enough thrust for a rotary ionic drone to be developed. We demonstrate here that a toroidal counter electrode can increase the RIE's performance by up to 7.8 times greater than in previous configurations (upper limit not determined). The RIE is designed with pin emitters extended on the trailing edge of a 12.6 cm two-blade plastic propeller placed above a toroidal counter-electrode which provided axial thrust up to 288.55 m Nat 23.15 N/m^2^, 4.2 m/s bulk airflow speed within the propeller plane, and 251 m^3^/h flow rate. The new design generates axial thrust due to the linear acceleration of ions between electrodes, and also due to the induced rotary motion of the propeller which captures the energy and momentum of ions accelerated in the propeller rotational plane. Thrust to power ratio can be measured by the ratio of voltage to current or propeller kinetic energy to power. A 4-RIE array matched the thrust (1 N) of a four-blade drone with similar blade size.

## Introduction

There is an increased interest in characterizing and harnessing the ionic wind for in-atmosphere propulsion^1–8^. The first demonstration of an ionic craft able to liftoff and carry its own power supply appears to have been performed by Krauss^9^ using negative polarity. More recently, Xu et al.^1^ demonstrated a plane/glider able to maintain flight using ionic wind generated in a positive polarity; a wirelessly powered flying ionic craft was also reported^10^. The ionic wind generated in atmospheric air can be produced by applying high voltage between two dissimilar electrodes and above the corona discharge threshold (but below the air breakdown value). Ions of the polarity applied to the emitter electrode are generated nearby sharp metal edges and within a very thin ionization layer in a fairly complex process^2^. Upon acceleration of the ions in the electric field between the electrodes, collisions with the adjacent neutral molecules happen and the ions eventually create an overall air movement from the emitter electrode to the counter electrode where they become neutralized. The electric field mediates the conversion of momentum and energy to the air movement and the electrode system. If the emitter can spin about an axis, ionic rotational motion can also be induced^11,12^. A rotational device with enhanced ionic wind emission was recently reported^13^.The device was designed with a propeller carrying pin emitter electrodes, concentrical cylindrical counter electrode and worked in negative polarity. Negative polarity was experimentally demonstrated to allow for an overall much larger thrust in air than positive polarity^14–16^. Negative corona discharges develop gradually into a breakdown while positive corona discharges develop abruptly into a breakdown. The breakdown voltage of an electrode configuration in negative polarity is larger than in positive polarity (in some of our RIE systems was almost double than for positive voltage breakdown value using the same electrode configuration). Therefore, the ions in negative polarity can be accelerated in a larger electric field intensitywithinthe voltage range not available to the positive polarity.

Using RIEs we show that slow rotation was produced in pure nitrogen in negative polarity when compared to atmospheric air^15^. This suggests that when compared to atmospheric air, little ionic wind is generated in pure nitrogen, conclusion also confirmed by other experimental work of Yan et al*.*^7^. In RIE systems, ionic propellers were able to spin and generate conventional axial thrust enough to lift off the high voltage shaft and fly freely for a while without carrying a power supply^14,15^. If enough axial thrust could be generated with rotary ionic engines, rotary ionic drones may be feasible. Such drones could potentially have low sound and thermal signature, simplicity of engine design, low cost, and no carbon emissions.

RIEs with coaxial contra-rotating propellers and zero angular momentum can also be built^16^. They would have some advantages over traditional atmospheric ion thruster configurations such as: kinetic energy storage by the propellers with additional flight stability added, smooth run even in non-uniform electric fields, and a relatively robust design when compared to the usage of extra thin and long wires for classic atmospheric thrustersconfigurations^16^. The first thrust measurements in RIEs were recently reported^13^. An axial thrust of 40 mN was obtained for a system with 12.6 cm two-blade propeller diameter with two pin emitters per blade and 17 cm diameter counter electrode cylinder. The RIE was operating at 0.33 mA, − 36 kV, and a thrust density of 1.76 N/m^2^. It was shown that the use of double coaxial propellers could lead to much larger thrust and thrust density if larger currents are available^13^. However, due to the fixed distance between the pin emitters and the counter electrode, a very significant limitation is imposed by the maximum voltage that can be applied before air breakdown occurs. Thrust density has been a recurrent issue with ionic crafts as they require a very large area for more meaningful thrust to be generated. Upper theoretical estimates, coming from a unidimensional model^17^, point to 20–30 N/m^2^ of maximum ionic thrust density that can be obtained in air at atmospheric pressure.

The present work addresses such limitations by changing the electrode configuration. A new counter electrode design is explored^18,19^. The propeller was coaxially placed above a toroidal counter electrode which intends to remove essential limitations of the original RIE configuration.

## Theoretical background

**Figure 1:**
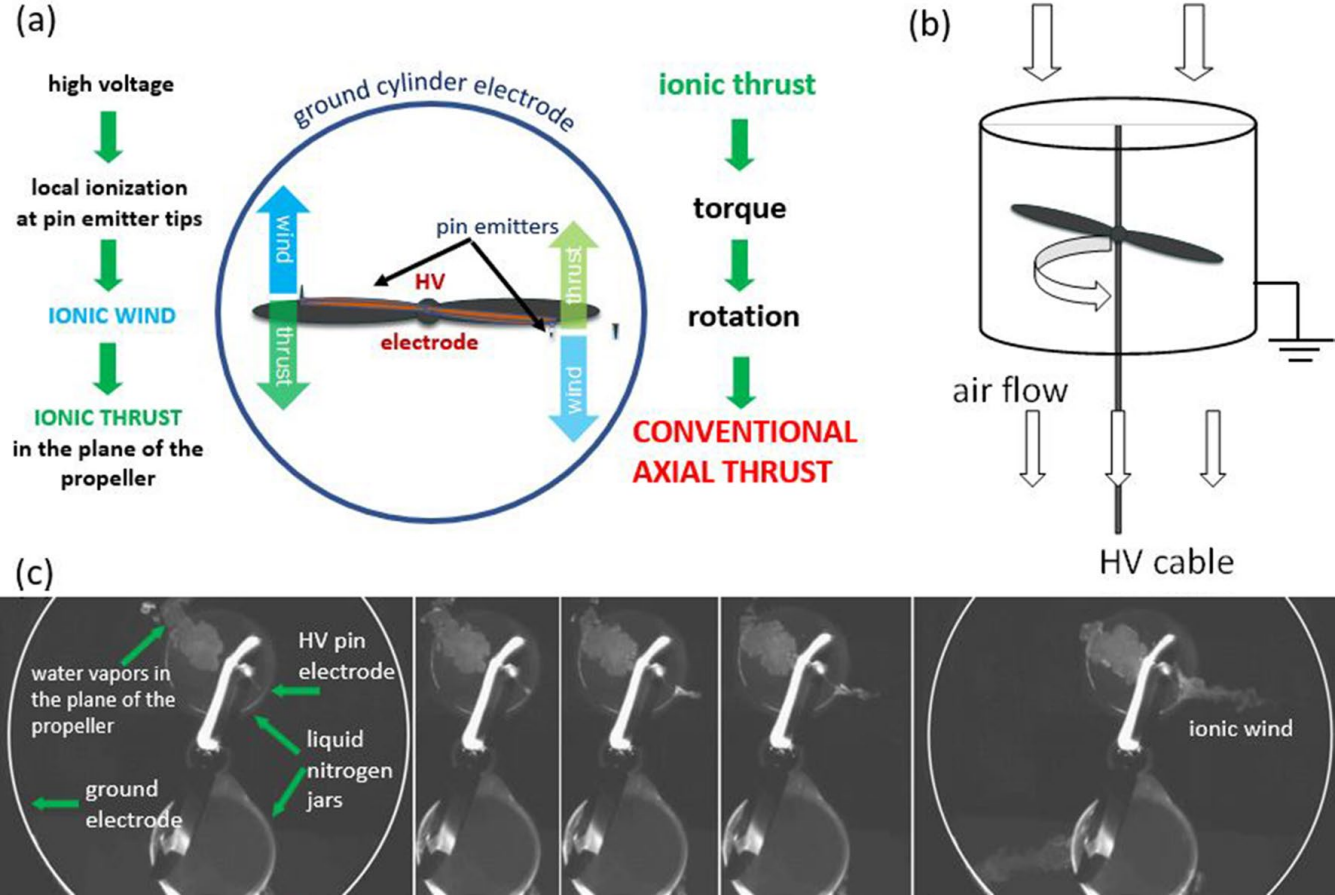
(**a**) Schematics of the mechanism of thrust in an ionic propeller-cylindrical counter electrode RIE (top view). (**b**) Side view of the RIE with cylindrical counter electrode and ionically induced air flow. (**c**) From left to right: 50ms image sequence of the ionic wind initiation in a RIE with cylindrical collector. A laser sheet was projected within the plane of the propeller rotation to visualize condensed water vapors induced by a liquid nitrogen container/jar placed below the ionic propeller. A Photron SA-X2 high-speed camera was used at 1000 fps.

The mechanism of thrust in the system is related to the ion emission and motion along the field lines during the corona discharge process. Such corona discharges in air result in multiple charge carrier species and dynamic space charge distributions which do not render themselves easy to precise modeling. This is due to the complexity of the processes involved in electrohydrodynamic flows of gas discharges. In the case of negative polarity applied to the pin emitters, the thrust results due to the negative ions produced in the close vicinity of the sharp edges of the pin electrodes. Although some positive ions are generated as well, they are quickly neutralized at the pin emitter tips where they are produced. The conceptual mechanism of thrust in a RIE with cylindrical counter electrode is suggested in Fig. 1. The high voltage applied between the electrodes generates a very intense electric field at the tip of the pin emitters, which leads to local ionization and production of mainly ions of the same kind to the polarity applied at the emitters. Within the thin ionization region, the coulombic repulsion between the negative pin tips and the ions is very large and leads to the propeller rotation. On the other hand, the moving ions in electric field convey momentum to the neutral air molecules generating ionic wind. Axial conventional thrust is produced due to the coulombic repulsion of the propeller blades and ions within the close region of ionization area. That further leads to torque and axial airflow and conventional axial thrust as the propeller spins (Fig. 1b). The very inception of the ionic wind in the RIE system was recorded on a high-speed camera (Photron SA-X2) using a special setup for visualization of the electrohydrodynamic flows (Fig. 1c). The wind starts at the very tip of the pin, apparently tangent to the pin, and further curves towards the cylindrical collector electrode.

The thrust in an ionic system can be calculated by accounting for all the electrical forces acting on the ions within a specific volume:

1 $$T=-\int \rho (\nabla V)dv$$

where *V* is the voltage potential and *ρ* is the charge density within the volume d*v*. The thrust to power ratio is a common measure for efficiency of propulsion systems. For RIEs with cylindrical counter electrode, the ratio was shown to be approximately proportional to the ratio of propeller rotational kinetic energy (KE) to power (P)^13,16^ at voltages much larger than the corona onset

2 $${\upeta }_{T}=\frac{T}{P}={\Lambda }_{\mathrm{E}}\frac{KE}{P}$$

where ${\Lambda }_{\mathrm{E}}$ is a constant. The thrust to power ratio was also shown to be proportional to the voltage to current ratio^13^

3 $${\upeta }_{T}=\frac{T}{P}={\Lambda }_{\mathrm{R}}\frac{V}{I}$$

where ${\Lambda }_{\mathrm{R}}$ is a constant.

**Figure 2:**
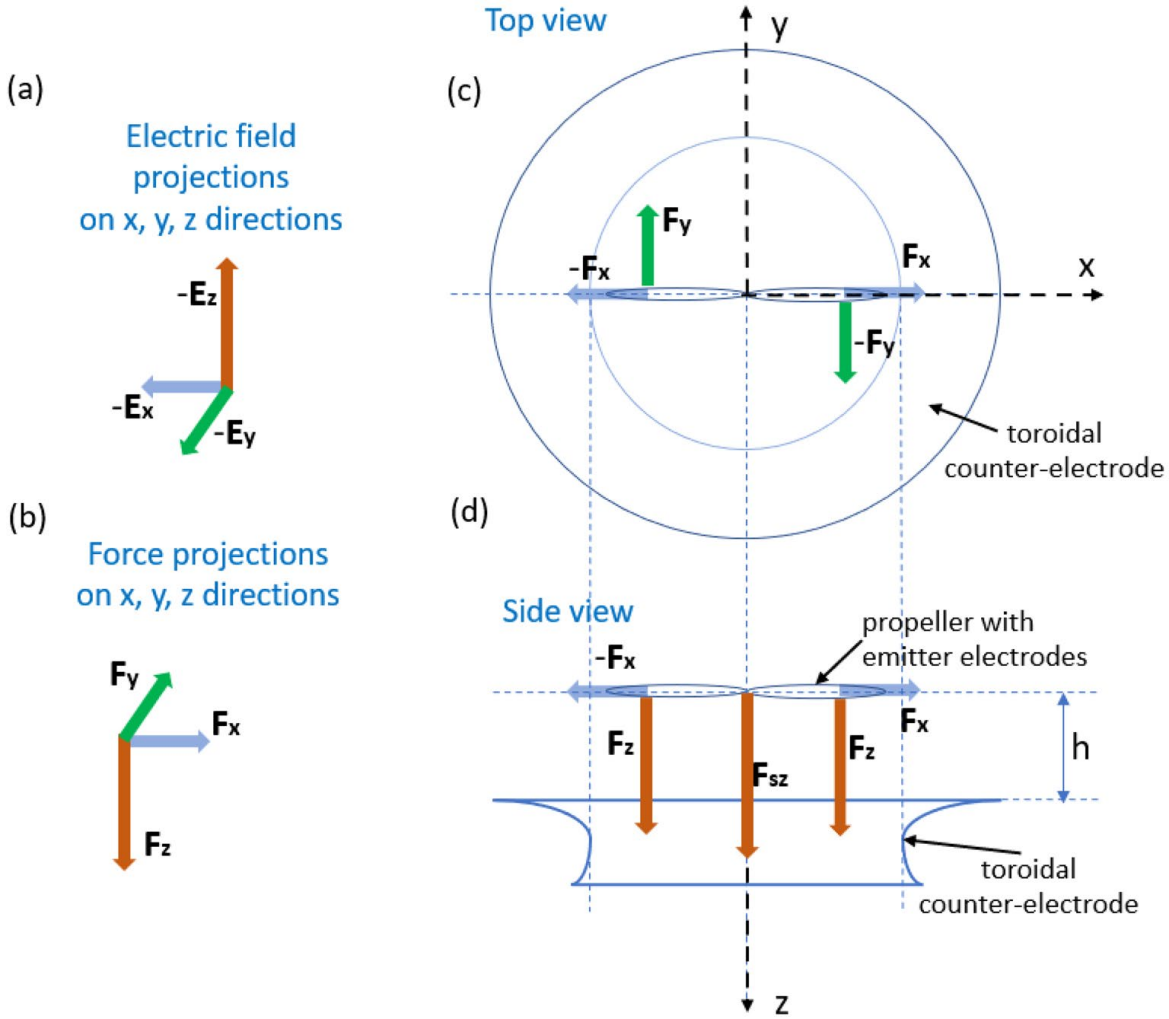
Axial thrust generation in a RIE with toroidal counter electrode. (**a**) Electric field projections at the emitter electrodes (**b**) Force acting on the ions projections at the emitter electrodes. (**c**) Top view of the RIE system. The propeller is coaxial to the toroidal counter electrode. (**d**) Side view of the RIE.

**Figure 3:**
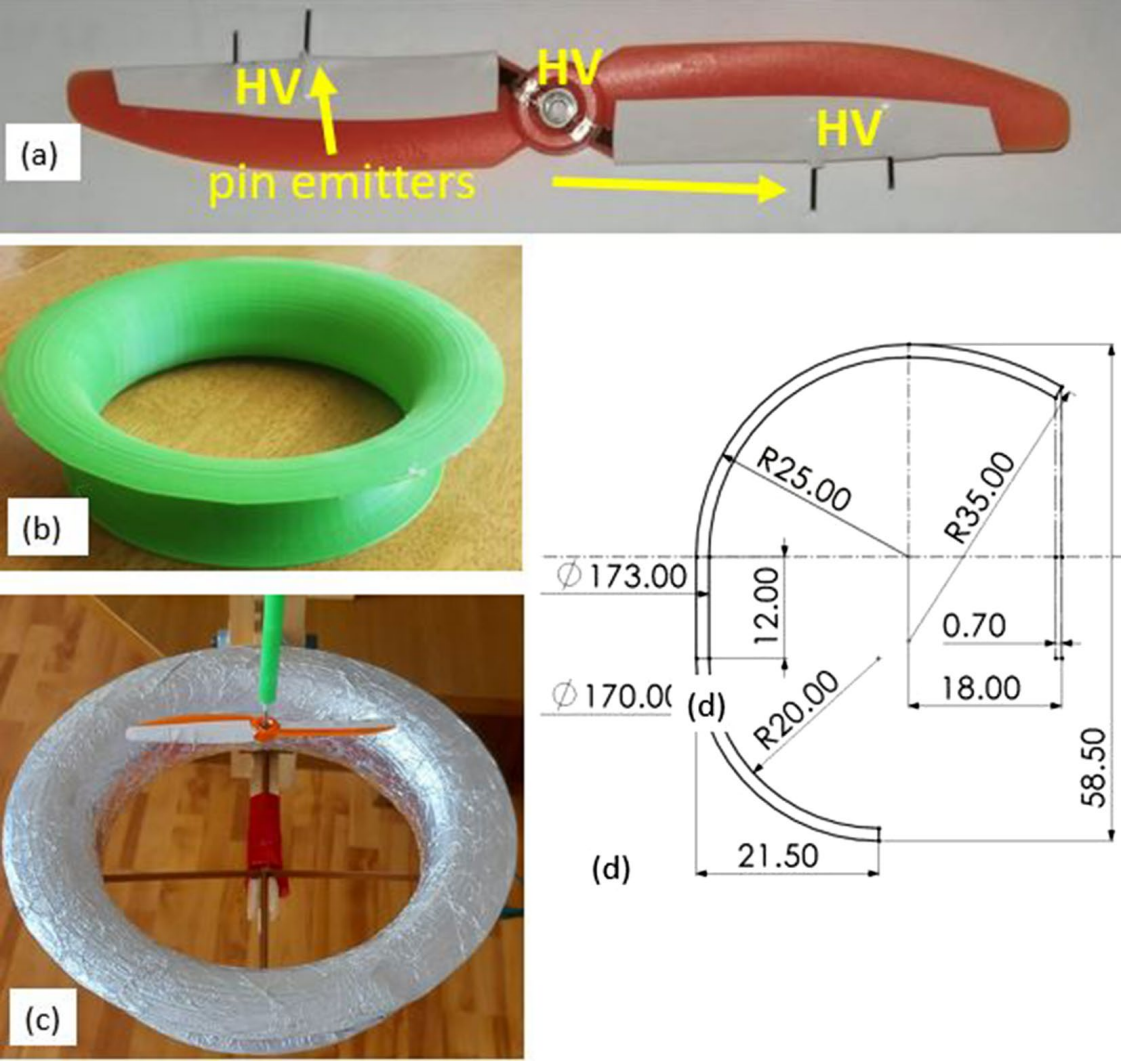
Toroidal RIE: (**a**) ionic propeller design with actual pin positioning (12.6 cm propeller diameter); the propeller is equipped with a ball bearing unit on which high voltage (HV) is applied and distributed via a copper tape to the pin emitters; (**b**) 3-D printed toroid; (**c**) toroidal RIE—the toroid is covered with aluminum foil to form the ground counter-electrode while the propeller is axially positioned above the toroid; (**d**) cross section details of the toroidal counter electrode design; all dimensions are given in mm.

Using a toroidal ground instead of a cylindrical one preserves the symmetry of the electric field during the rotational motion of the propeller equipped with pin emitters. Experimental work presented here shows that RIEs with toroidal ground counter electrodes (Figs. 2 and 3) appear to have significant advantages in helping the system achieve more axial thrust than RIEs with cylindrical grounds. The ionic thrust was demonstrated in unidimensional models (also experimentally in certain electrode configurations) to be directly proportional to the corona current^19–22^. However, as the ionic current has a three-dimensional distribution and not necessarily aligned in the direction of useful thrust, such relation can only be a rough approximation in arbitrary configurations. Figure 2 shows the schematics of a RIE with pin emitters on a propeller placed above a toroidal ground counter electrode.

At the very core of the ionic thrust is the conservation of momentum of the electrode–air–charge carriers system which is mediated by the electric field. As the field lines are generated in space, there are three components of the electric field along the Ox, Oy, Oz, axes in which ions can and do move. Therefore, the associated ionic thrust has components along those axes. With a 3-D signature, the overall ionic thrust emerges as the superposition of the three components along the axes. In the case of the ionic propeller–toroid system (Fig. 2), the axially generated thrust combines the mechanism of thrust for ionic propeller–cylinder system (Fig. 1) to the classic linear momentum conservation in systems like pin-to-wire, wire-to-cylinder, or wire-to-airfoil systems^1,23,24^.

The electric field generated above the toroid can be viewed as superposition of the field produced by rings of charges making up the toroid. An intense field is then generated at the tip of the emitter electrodes. Ions are emitted in the very proximity of the pin tips and are subject to the electric field produced by the electrodes. As pin emitters are extending on the trailing edge of the blades, emitted ions will have an induced motion along the electric field projections: **E**_**x**_—along the blade, **E**_**y**_—perpendicular to the blade and in the propeller rotational plane, and **E**_**z**_—parallel to the propeller axis of rotation. The corresponding electrical forces, **F**_**x**_, **F**_**y**_, and **F**_**z**_, generate thrust and eventually rotational motion of the propellers due to the conservation of linear and angular momentum^15^. **F**_**z**_ leads to linear acceleration of ions between electrodes and to the generation of upwards axial thrust. **F**_**y**_ leads to torque exerted on the blades, which spins the propeller and generates upwards axial conventional thrust. **F**_**x**_ does not lead to useful axial thrust as it is directed along the blade and in opposite directions for the two blades. Due to the field lines orientation above the toroid, the relative magnitude of **F**_**x**_ is estimated to be small by comparison to the other force projections. The overall axial thrust is therefore a superposition of the thrust associated to **F**_**z**_ and **F**_**y**_ (while it spins the propeller and so generates conventional axial thrust). The propeller shaft acts as a pin emitter above the toroid and contributes to the axial thrust. The ions emitted at the central shaft **F**_**sz**_ also lead to axial thrust superimposed on the axial thrust effects of the pin emitters on the blades.Therefore, the overall axial thrust is a summation of the conventional thrust due to the rotation of the propeller (induced by the ionic thrust in the plane of the propeller—**F**_**y**_), and axial thrust (**F**_**z**_ + **F**_**sz**_,) resulting from the field-induced motion of ions in the axial direction between electrodes (ions generated both by the pin emitters on the blades and also from the central shaft).

RIEs with cylindrical counter electrode (Fig. 1) have strong limitations in generating axial thrust partly due to the finite distance between emitter electrodes and cylinder walls. This leads to a maximum voltage that can be applied before gas breakdown occurs. This limitation is avoided by using a toroidal counter electrode configuration, as the propeller with emitters can always be elevated at an appropriate distance above the toroid so that a breakdown does not occur.

Given that field lines direction and density change with the propeller position above the toroid, **F**_**z**_, **F**_**sz**_ and **F**_**y**_ and their associated axial thrust also vary, so allowing for an optimal propeller height at which axial thrust is maximum for a given applied voltage (and above the corona onset).

## Results and discussion

### Toroidal RIE performance

**Figure 4:**
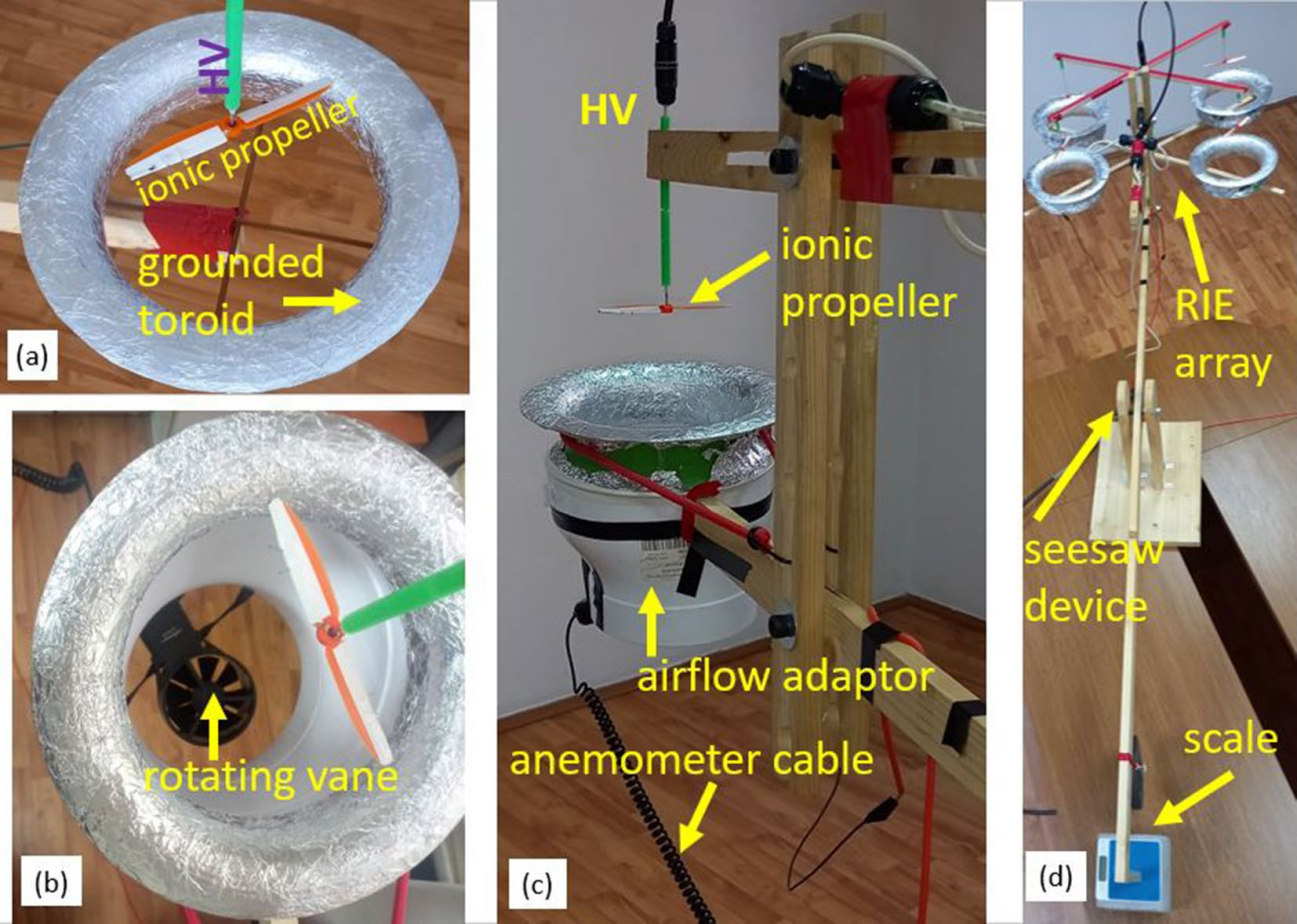
Toroidal RIE and measuring setup: (**a**) Toroidal RIE mounted on the measuring device; negative high voltage is fed from the shaft above the toroidal ground collector; (**b**) setup used for measuring the airflow speed and estimation of the volume flow rate; an anemometer with a rotating vane and a plastic adaptor for focusing flow are used as shown; (**c**) details of the ionic system mounted on the thrust measuring seesaw device (**d**) 4-RIE array mounted on the seesaw measuring device; at the opposite end there is a balancing weight and an electronic scale measuring the overall axial ionic thrust.

**Figure 5:**
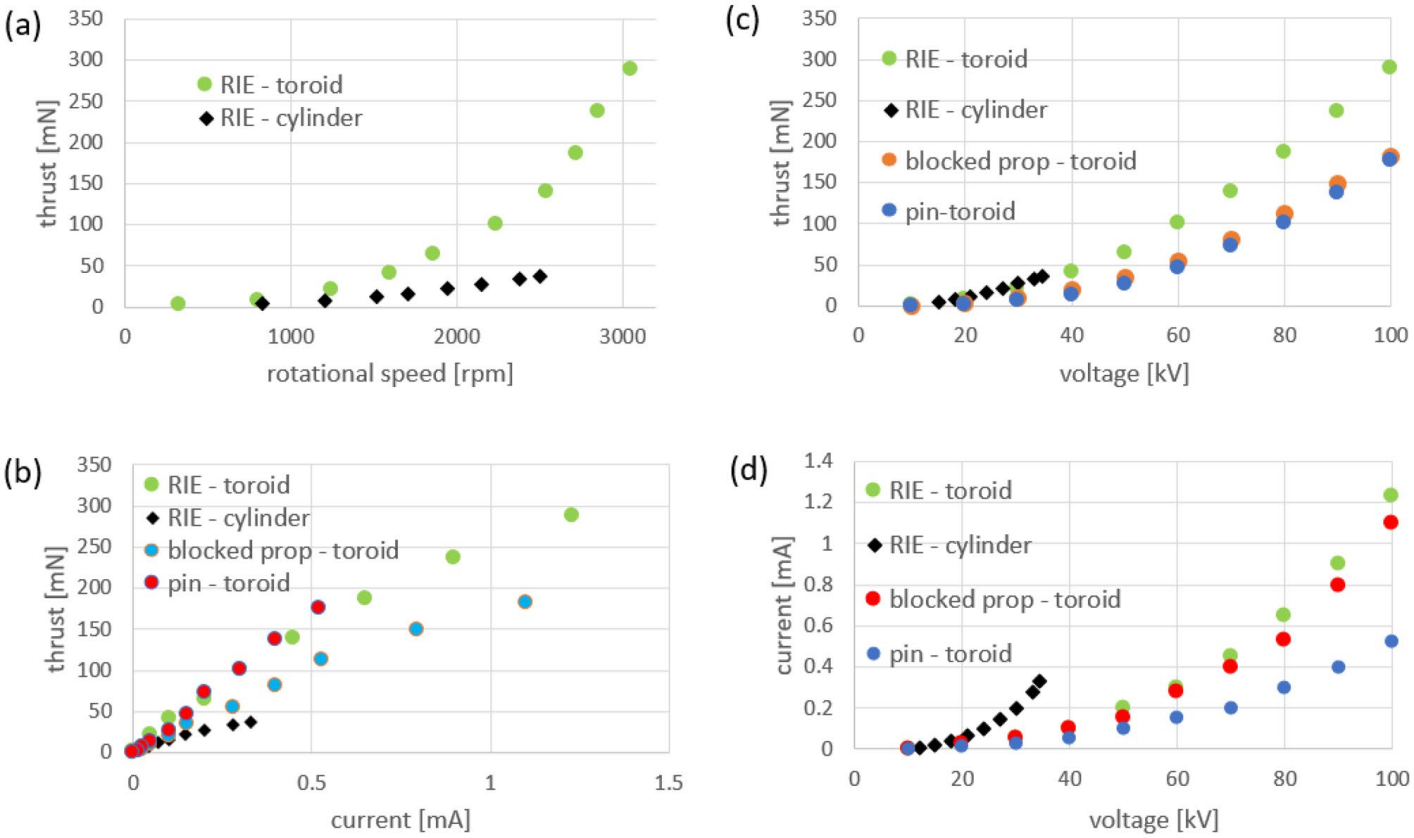
Thrust measurements in ionic systems with toroidal and cylindrical counter electrodes (the toroid minimum diameter is 17 cm and equal to the cylinder electrode). (**a**) Variation of the ionic axial thrust with rotational speed in RIEs with toroidal and cylindrical counter electrodes (**b**) Variation of the ionic axial thrust with current in RIEs with toroidal and cylindrical counter electrodes; additional comparison is made for systems with a blocked ionic propeller and for systems using the shaft/pin as emitter. (**c**) Variation of the ionic axial thrust with voltage (**d**) Current–voltage characteristics of the studied configurations.

Optimized RIEs with pin emitters set on a 12.6 cm two-blade propeller (Fig. 3a) with toroidal grounds were tested for performance. The propeller can spin on a high voltage shaft above a toroidal counter electrode(Figs. 3c and 4a–c), and it generates vertical axial thrust when high voltage above the corona onset is applied. The minimum inner diameter of the counter electrode was found empirically to be 17 cm (larger or smaller diameters led to decreased axial thrust). Negative high voltage up to 100 kV was applied to the pin emitters and the axial thrust generated was measured with an electronic scale placed on the opposite side of a seesaw system on which the RIE was mounted (Fig. 4d). Maximum thrust of 288.55mN was obtained for the better optimized RIEs as shown in Fig. 5a–c.The values plotted are the average of five independent measurements with a standard deviation of 0.216 mN (or 0.734% of the average). The corresponding thrust density was 23.15 N/m^2^ if the propeller diameter (12.6 cm) is considered or 12.72 N/m^2^ if the inner diameter of the toroid (17 cm) is used in the calculation.

### Comparison of RIEs with toroidal and cylindrical counter electrodes

The thrust increases with rotational speed of the propeller (Fig. 5a), current (Fig. 5b), and voltage (Fig. 5c). A comparison of the ionic thrust obtained with toroidal RIE versus cylindrical RIE designed with the same propeller and pin emitters as thetoroidal RIE and the same inner diameter (17 cm) of the cylinder counter electrode is shown in Fig. 5a. While the thrust increases approximately linearly with rotational speed for the cylindrical RIE, it increases significantly more than linearly for the toroidal RIE.

Additional testing was performed using the toroidal RIE involving the original system but having the propeller blocked so no spinning was possible. Also, in a different experiment, the propeller was removed, with only the high voltage shaft remaining above the toroidal ground. Comparison charts are given in Fig. 5b–d. The variation of axial thrust with current is given in Fig. 5b for all the systems studied. The cylindrical RIE provides the smallest thrust at any given current value. If the propeller is locked and cannot spin in a toroidal RIE, the resultant axial thrust is significantly smaller than for the case the propeller is free to spin but above the values recorded for cylindrical RIE. If the propeller is removed from the toroidal RIE, a pin (emitter)—toroid (counter electrode) system is formed, which at a given current provides marginally more thrust than for the system with the propeller spinning. However, the current does not reach the levels of the toroidal RIE. The maximum thrust for toroidal RIE (288.55) is 63.8% larger than for pin/shaft—toroid configuration (176.1 mN). Both the pin–toroid and the locked propeller–toroid systems provide about the same maximum thrust (181.4 mN and 176.1 mN correspondingly). However, the locked propeller system runs at a current and power (1.23 mA, 123 W) much larger than the pin-toroid system (0.52 mA, 52 W).

A different view of the examined atmospheric ionic thrusters relative performance is given in Fig. 5c. At a given voltage, the largest thrust is given by the cylindrical RIE. Nevertheless, the voltage that can be applied before breakdown is limited by the small distance between the inner surface of the cylinder counter electrode and the pin emitters on the blades placed coaxially and inside the cylinder. This limitation renders the cylindrical RIE to overall generate very small thrust by comparison to the toroidal RIE where voltage breakdown can always be avoided by elevating the propeller more above the counter electrode (which eventually brings the electrical field below the breakdown value). For the present comparison, the maximum value for the toroidal RIE is 778.1% larger than the thrust given by the cylindrical RIE. The thrust provided by the locked propeller and the pin/shaft–toroid systems are very close at any given voltage (with the locked propeller thrust marginally larger) and significantly smaller than for the toroidal RIE (37% smaller at 100 kV and increasingly smaller at lower voltages—by 63.5% at 20 kV). The increase in the thrust of the toroidal RIE with propeller spinning versus the pin/shaft–toroid system at a given voltage is 63.8% larger at 100 kV and increasing up to 429.1% at 20 kV. The thrust generated by the locked propeller versus the pin/shaft–toroid system is marginally larger at any voltage. It demonstrates that the additional pin emitters on the blades do not contribute much to additional axial thrust when the propeller cannot spin. The addition is only 3.2% at 100 kV and increases at lower voltages. At 20 kV the contribution to the original axial thrust provided by the pin-toroid system only is 93%. However, if the propeller is allowed to spin, the contribution amounts to an additional 429.1% at 20 kV.

Although the thrust of the toroidal RIE and the locked propeller system (Fig. 5c) are significantly different (the toroidal RIE was 57.8% larger at 100 kV and with an increasing percentage relative to the locked propeller system at lower voltages), the corresponding currents at a given voltage present much smaller change (11.8% larger at 100 kV)—see Fig. 5d. Once the propeller is allowed to spin freely, the thrust jumps up as the ion momentum in the plane of the propeller rotation is captured and converted into rotational motion and eventually into additional conventional axial thrust. A comparison of the current–voltage characteristics of the systems studied is given in Fig. 5d. At a given voltage, the cylindrical RIE gives the largest current and thrust. At 25 kV it provides about 25% more thrust than the toroidal RIE. However, the difference is reduced with the increase in voltage. Moreover, the breakdown voltage is reached quickly, which caps the corona current and the obtainable thrust in the cylindrical RIE.

**Figure 6:**
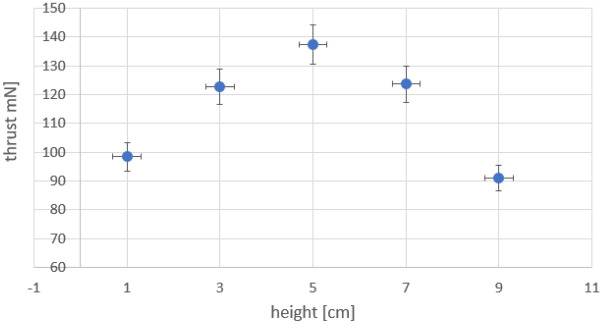
Sample variation of the axial thrust with the height above the propeller at − 98 kV for a RIE with toroidal collector. Error bars are 3 mm height positioning error and 5% estimated measurement thrust error.

The propeller holding the pin emitters can be placed coaxially at different heights above the toroidal ground (Fig. 3c). In RIE with toroidal counter electrode the axial thrust is the summation of conventional propeller thrust and the thrust resulting from the axially accelerated ions. The two component values and ratio change with the height of the propeller above. The higher the propeller is positioned above the toroid, the smaller the electric field component **E**_**y**_ (Fig. 2) to accelerate ions in plane of the propeller, the less torque and conventional thrust for a given voltage. The axial thrust (**F**_**z**_ + **F**_**sz**_), decreases as well but at a slower rate. Due to the differences in the rates of change in the two axial thrust components, an optimum height with a maximum thrust is expected be present at any given voltage above corona onset. Experimental results show the actual presence of a maximum axial thrust when the voltage is kept constant but the propeller height above the toroid is varied. Such an example is shown in Fig. 6 for a test at − 98 kV for one of our toroidal RIE units. The thrust is controlled by the height in this example by about 40–50% relative to its maximum (not precisely identified in this example) both at lower and higher heights than what turns to be an optimum height for the applied voltage and electrode configuration. Heights greater than the optimum lead to lower thrust due to the generated lower intensity of the electric field and accordingly less ion acceleration and resulted thrust.The comparison in Fig. 5c,d (of the pin–toroid and the locked propeller–toroid systems) suggests that very little axial thrust (**F**_**z**_) is generated by the pin electrode-generated ions although the ions eventually move axially and reach the toroid collector. This would be consistent with the assumption that the ionic thrust results from the coulombic interaction of the pin emitters and the ions within a very limited region of the ionization zone and mostly within the propeller plane.

**Figure 7:**
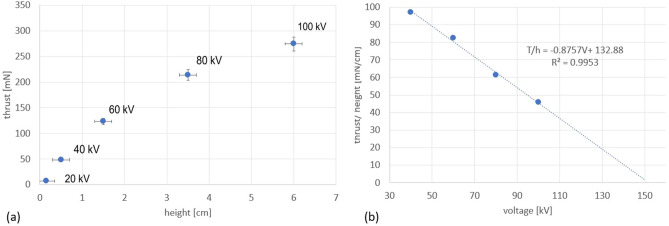
(**a**) Maximum RIE axial thrust dependance on the propeller axial height above the toroidal counter electrode at various voltages. Error bars are 3 mm height positioning error and 5% estimated measurement thrust error. (**b**) Variation of the axial thrust per cm height for a 20 kV increase in the applied voltage and positioning of the propeller above toroid at the optimal height (maximum thrust for the applied voltage).

A series of measurements were performed at different voltages (increments of 20 kV) and optimum height for each voltage. The results presented in Fig. 7a show an increase in the axial thrust with voltage and optimum height. As the voltage is increased, a greater height is needed to reach the optimal thrust. However, the rate at which the thrust per optimal height increases with the increase in voltage (Fig. 7b) is diminishing. A linear decrease in this rate is suggested by the chart and pointing to a voltage at about 150 kV where any additional gain in the axial thrust will no longer be present.

### Toroidal RIE arrays

Additional testing was performed on toroidal RIEs optimized individually to a maximum thrust of about 250 mN at 100 kV. A series of 10 measurements were performed for a single unit RIE to assess the bulk speed of the airflow within the region of the toroid exit area. An anemometer with a rotating vane and the setup shown in Fig. 4b,c was used to measure the speed of the exit airflow at − 100 kV, 1.1 mA and 3057 rpm propeller speed. The measured average speed was 3.96 m/s with a standard deviation of 0.098 m/s (2.47%) at the exit of the air flow adaptor (of 7.5 cm radius). The flow rate corresponds to 251 ± 6.22 m^3^/h. As the narrowest radius of the toroid is 8.5 cm and assuming the same volume flow rate as for the exit of the airflow adapter, an airflow speed of 3.083 ± 0.387 m/s would result at the toroid exit. The ionic thrust was also measured for each of the 10 anemometer measurements using the seesaw device and the precision electronic scale (Fig. 4d). The average of the 10 measurements provided a scale reading of 264.25 mN with a standard deviation of 2.04 mN (0.76%). The axial ionic thrust (*T*) can also be calculated from the fundamental force relation:

4 $$T=\frac{d(mv)}{dt}=\frac{d(\rho Vv)}{dt}=\rho A{v}^{2}$$

and

5 $$v=\sqrt{\frac{T}{\rho\uppi {r}^{2}}}$$

where *m* is the mass of the airflow through a transversal cross-section *A*, $\rho$ is the air density at room temperature, *v* is the speed of the airflow in the cross-section *A*, *r* is the radius of the circular area *A*. Using the average value of the measured thrust (0.26425 N), the exit toroid radius (8.5 cm) and 1.2 kg/m^3^ for the air density at room temperature, the estimated bulk speed of the airflow gives 3.11 m/s—according to relation (5). The corresponding flow rate through the toroid exit cross-section is 253.99 m^3^/h or 1.19% larger than the value resulting from anenometer measurements. The value airflow speed is also within 1% difference from the estimated speed for the same toroid cross-section resulting from the anenometer measurements (3.083 m/s). If the cross-section area *A* is taken at the propeller level (*r* = 6.3 cm), a bulk airflow speed of 4.2 ± 0.032 m/s results.

**Figure 8:**
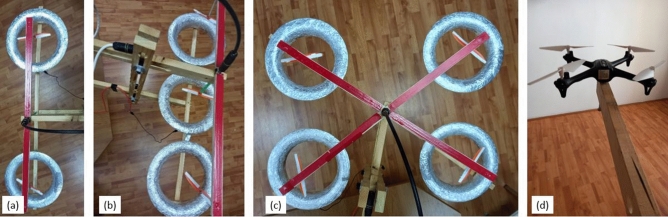
Top view of theRIE array setup mounts for ionic thrust measurements. (**a**) two-RIE toroidal array (**b**) three-RIE toroidal (**c**) four-RIE toroidal array. (**d**) mounted four-blade X15A Guangdong Syma drone; the drone generated a maximum lifting force very close the maximum thrust recorded for the 4-RIE array.

**Figure 9:**
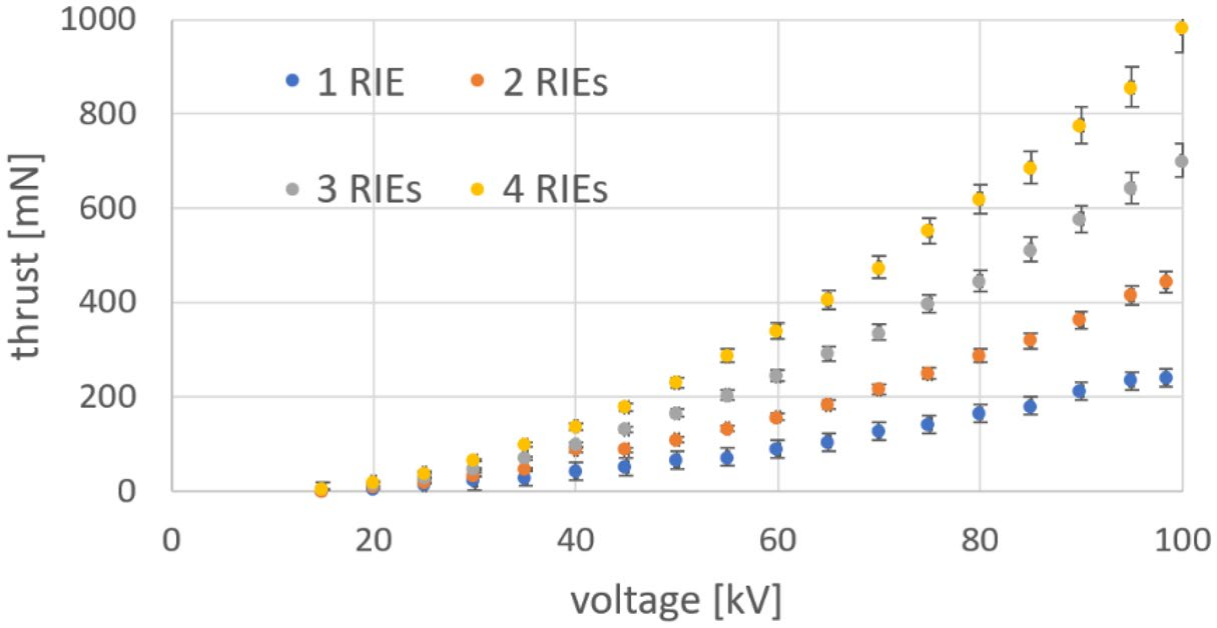
The toroidal axial thrust dependence on the voltage and the number of parallel-connected units/toroidal RIEs used. The error bars represent potential measurement errors related to setting the voltage (up to 0.2 kV) and 5% thrust errors (due to the mounting of the RIE on the measuring device and electronic scale readings.

Toroidal RIE systems with one to 4 units were tested for potential interference in their performance when working in an assembly of units. They were mounted on a support structure and the joint axial thrust was measured with the same procedure used for a single unit. RIE arrays with 2 to 4 units were mounted on the measuring system as shown in Fig. 8 (also Fig. 4d). The thrust dependence on voltage and the number of units used is presented in Fig. 8. Small variations of thrust were noticed with the number of toroidal RIEs which can be attributed to the fact that the RIEs were not perfectly identical. Although a dependence on the arrangements of the array may be estimated at large air flow speeds it may not be significant at relatively low airflow speed around 3.11 m/s estimated in our systems in the exit area. We thought that some effect may be apparent due to the electric field interference from one system to another. However, such effect was not large enough to be clearly observed whether the units were adjacent or not. The axial thrust of the multi RIE system appears to overall scale linearly with the number of RIE units used as shown in Fig. 9. Any differences observed in the measurements may be attributed to inherent slight variations in the RIE designs and test conditions.

The thrust of the 4-RIE array (Fig. 4c) was compared to a four-blade X15A Guangdong Syma drone which uses similar propeller blade diameter as the RIE array. The drone was mounted on the same seesaw device (Fig. 4d) used for testing RIEs. A maximum of 3300 rpm was measured for the blade speed and a 100 g-force (981 mN) for the overall thrust. The thrust X15A drone provided was essentially the same as the maximum thrust recorded for the 4-RIE array (Fig. 9).

### Measures for thrust to power ratio

**Figure 10:**
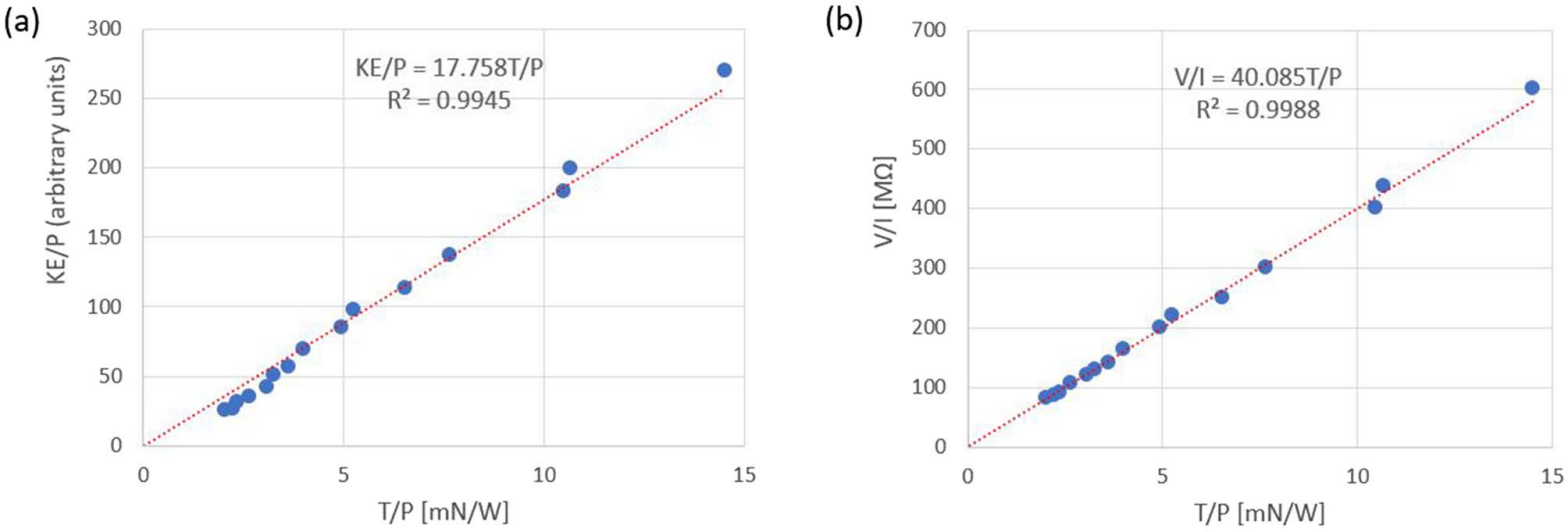
(**a**) Experimentally observed direct proportionality of the thrust to power ratio (T/P) and the propeller kinetic energy to power ratio (KE/P). (**b**) Experimentally observed direct proportionality of the T/P ratio and the voltage to current (V/I) ratio.

A direct proportionality of the thrust to power ratio (T/P) and the propeller kinetic energy to power ratio (KE/P) at voltages much larger than corona onset voltage was observed for toroidal RIEs. The scaled kinetic energy to power ratio (KE/P) for our experimental data is plotted in Fig. 10a against the thrust to power ratio. A linear regression line crossing the origin of axes has a coefficient of determination of 0.9944 which demonstrates the two plotted ratios are in a very close direct proportionality relationship as given by relation (2).

The experimental V/I ratios was also plotted against the thrust to power ratio in Fig. 10b. Data regression was conducted on a line crossing the origin of the coordinates. The coefficient of determination (very close to value of 1) shows again that the experimental data agrees with a direct proportionality of the two plotted ratios as relation (3) suggests. Hence, the thrust to power ratio appears to be well measured for toroidal RIEs by the KE/P or V/I ratios (less a proportionality constant).

## Conclusion

A new RIE with toroidal counter electrode was demonstrated. A maximum axial thrust of 288.55 mN/RIE at 23.15 N/m^2^ was obtainedat an estimated volume flow rate of 251 m^3^/h. The thrust density value is within the upper limit of 20–30 N/m^2^ predicted by theoretical models. The value was obtained for a sizable surface. It may be possible that at higher voltages larger thrust and thrust densities could be achieved. The comparison of the new system to the cylindrical RIE shows an increase in the available thrust up to 778.1%. The new design allows for capturing the thrust related to ions linearly accelerated in-between the electrodes and also within the plane of propeller rotation. The proportionality of the ionic thrust to the corona current is valid for a unidimensional model. However, the ionic current has a tridimensional distribution. Hence, the current density would allow for better characterization of the thrust in relation to the ionic current generated. The experiments showed that toroidal RIEs with freely spinning or locked propeller present only small differences in the level of currents but major ones in terms of axial thrust. The thrust obtained with arrays of toroidal RIEs was overall proportional to the number or RIEs proving the scalability of the systems. An experimental assessment of the thrust to power ratio was performed indicating that propeller kinetic energy to power and voltage to current ratios are suitable measures for it. The new RIE designs allowed for a very substantial increase in the axial thrust with respect to previous RIEs. The 4-RIE array produced a maximum thrust of almost 1 N (or about 100 g-force), similar to the maximum thrust of the commercial four-blade X15A Guangdong Syma drone (using similar diameter propellers like our RIE array).

## Methods

RIEs with toroidal grounds were built with two-blade plastic propellers of 12.6 cm diameter and 1.87 g mass. The propellers were equipped with pin emitters as described in^16^ and shown in Fig. 3a. Copper tape was used to connect the propeller ball bearing to the central shaft and with two 0.4 cm regular pin emitters per blade extending on the blade trailing edge. The copper tape connections are covered with electrical tape which limits the corona current losses. The propeller was placed on a negative high voltage shaft coaxially and above a toroidal ground counter electrode. A plastic shape of the toroidal counter electrode was 3-D printed (Fig. 3b). Various shapes were tested in search for a more optimal shape. The minimum diameter of the toroid was found optimal at 17 cm diameter, which is similar to the diameter used with the same type of propellers for RIEs with cylindrical ground^16^. Eventually, other specific dimensions were found to work well with the designed ionic propeller (Fig. 3d). Once printed, the toroid was covered in aluminum foil to produce the ground collector electrode (Fig. 3c).

The negative high voltage was applied to the RIE emitter electrodes from a Glassman power source PS/KT100R20-220 (± 100 kV, 20MA). Rotational speed of the propeller was measured with a PCE-OM15 digital stroboscope tachometer with a response time of 0.3 s, 0.1 fpm resolution below 1000 fpm speed, and 1 fpm resolution above 1000 fpm. High speed imaging (Fig. 1c) was performed at 1000 fps with a Photron SA-X2 high-speed camera. Thrust measurements were performed with a two-meter long “seesaw” manufactured device which keeps the electronic scale far enough to cancel the electric effect from the high voltage testing site^16^. The device allows for the separation between the intense electric field needed for the ionic wind and the thrust measuring devices—where no intense electric field should be present. The length of the seesaw arm was chosen experimentally, and with a large margin, so that the measuring electronic scale measurements are not influenced by the electric field generated in the high voltage region. The system was previously tested at the highest voltage available (100 kV) to fully verify that no data corruption is induced when using the scale with or without high voltage. Physical details of the measuring setup can be seen in Fig. 4d. The seesaw structure balances around a central pivot. At one end the ionic system is mounted and powered with high voltage while at the opposite end a vertical arm extension rests on an Mayam electronic scale able to measure up to 500 g-force with a 0.001 g-force accuracy. The scale was zeroed every time the setup was ready for thrust measurements and before the high voltage was applied. When RIE is mounted on the structure, the arm length on the high voltage area/ RIE and the one on the electronic scale are equal hence facilitating direct readings of the thrust in g-force on the scale. The mounting of the RIE is more visible in Fig. 4c. The balancing arm was designed with an additional structure to facilitate easy mounting of the RIE on the arm and also variable positioning of the propeller above the toroid collector. Additional supporting structures were designed for each of the 2–4 RIE arrays to be tested as shown in Fig. 8. Direct airflow speed measurements were performed using a MulticompPro anemometer with a rotating vane (Fig. 4b,c). The anemometer has a 0.4–30 m/s measurable wind speed range and − 10 °C to + 50 °C allowable temperatures. A plastic adaptor was used to better capture the airflow from the toroid exit and also to protect the anemometer from malfunctioning or getting destroyed in very intense electric fields. The adaptor has an exit diameter of 15 cm (Supplementary video).

## Supplementary Information

Supplementary Video 1.

## Data Availability

The authors declare that the data supporting the findings of this study are available within the paper. All additional raw and derived data that support the claims within this study are available from the corresponding authors upon reasonable request.

## References

[1] Xu H (2018). Flight of an aeroplane with solid-state propulsion. Nature.

[2] Park S, Cvelbar U, Choe W (2018). The creation of electric wind due to the electrohydrodynamic force. Nat. Commun..

[3] Haofeng X, Yiou H, Barrett SRH (2019). A dielectric barrier discharge ion source increases thrust and efficiency of electroaerodynamic propulsion. Appl. Phys. Lett..

[4] Du S (2018). Laser guided ionic wind. Sci. Rep..

[5] Sato S (2019). Successively accelerated ionic wind with integrated dielectric-barrier-discharge plasma actuator for low-voltage operation. Sci. Rep..

[6] Johnson MJ, Go DB (2017). Recent advances in electrohydrodynamic pumps operated by ionic winds: A review. Plasma Sources Sci. Technol..

[7] Yan P (2016). An experimental study on the effects of temperature and pressure on negative corona discharge in high-temperature ESPs. Appl. Energy.

[8] Fylladitakis ED, Theodoridis MP, Moronis AX (2014). Review on the history, research, and applications of electrohydrodynamics. IEEE Trans. Plasma Sci..

[9] Krauss, E. *Self-Contained Ion Powered Aircraft*. *US Patent 10*,119, 527 B2 (2018) (Provisional Application No. 62/034394, 2014).

[10] Khomich VY, Rebrov IE (2018). In-atmosphere electrohydrodynamic propulsion aircraft with wireless supply onboard. J. Electrostat..

[11] Wilson B (1760). Farther experiments in electricity. Phil. Trans. R. Soc. Lond..

[12] Robinson M (1962). A history of the electric wind. Am. J. Phys..

[13] Ieta, A. & Chirita M. First thrust measurements in ionic multi-propeller rotational engines. https://www.researchsquare.com/article/rs-2241697/v1 (2022).

[14] Ieta, A. *An Electrohydrodynamic Rotational Device*. WO US CA US20210143722A1. (Research Foundation for The State University Of New York, 2021). Priority 2018–05–21, Filed 2019–05–21. Accessed 13 May 2021.

[15] Ieta A, Chirita M (2019). Electrohydrodynamic propeller for in-atmosphere propulsion: Rotational device first flight. J. Electrostat..

[16] Chirita M, Ieta A (2022). First rotary ionic engine with contra-rotating propellers. J. Prop. Power.

[17] Pekker L, Young M (2011). Model of ideal electrohydrodynamic thruster. J. Prop. Power.

[18] Ieta, A. & Chirita, M. Toroidal counter-electrode for ionic thruster. *Patent application,* SUNY RF case #: 230-2218.

[19] Chirita M. Toroidal counter-electrode for ionic thruster. *Patent application*, INCEMCT, OSIM Nr. A/00268/17.05.2022.

[20] Wilson, J., Perkins, H. D. & Thompson, W. K. An investigation of ionic wind propulsion. *NASA/TM* 215822, https://ntrs.nasa.gov/api/citations/20100000021/downloads/20100000021.pdf (2009).

[21] Moreau E, Benard N, Lan-Sun-Luk JD, Chabriat JP (2013). Electrohydrodynamic force produced by a wire-to-cylinder corona discharge in air atmospheric pressure. J. Phys. D.

[22] Ianconescu R, Sohar CD, Mudrik M (2011). An analysis of the Brown–Biefeld effect. J. Electrostat..

[23] Belan M (2021). A parametric study of electrodes geometries for atmospheric electrohydrodynamic propulsion. J. Electrostat..

[24] Belan M, Terenzia R, Trovatoa S, Usuellia D (2022). Effects of the emitters density on the performance of an atmospheric ionic thruster. J. Electrostat..

